# To Shunt or Not to Shunt Patients with Idiopathic Normal Pressure Hydrocephalus? A Reappraisal of an Old Question

**DOI:** 10.3390/jcm9124120

**Published:** 2020-12-21

**Authors:** Juan Sahuquillo, Maria A. Poca

**Affiliations:** 1 Department of Neurosurgery, Vall d’Hebron University Hospital, Vall d’Hebron Barcelona Hospital Campus, Passeig Vall d’Hebron 119-129, 08035 Barcelona, Spain; 2 Neurotrauma and Neurosurgery Research Unit, Vall d’Hebron Institut de Recerca (VHIR), Passeig Vall d’Hebron 119-129, 08035 Barcelona, Spain; 3 Universitat Autònoma de Barcelona, 08193 Bellaterra, Spain

The possibility that the cerebral ventricles can dilate without any increase in the pressure of the cerebrospinal fluid (CSF) was recognized in 1935 by Penfield who reported a patient in whom “…the cerebrospinal fluid spaces are closed and the ventricles progressively enlarge without the measured intraventricular pressure rising above 150 to 200 mm of water” [1]. However, the normal pressure hydrocephalus (NPH) syndrome was first described by Hakim in 1964 in his PhD thesis and by Hakim and Adams in 1965 [2,3]. In their seminal paper, Hakim and Adams reported on three patients with ventriculomegaly and severe dementia—two posttraumatic and one idiopathic—associated with a CSF lumbar pressure below 180 mmH_2_O (14 mmHg) that significantly improved after implanting a ventriculoatrial shunt [2,3]. In a second paper published the same year, they added three similar patients and proposed the term NPH to define a syndrome in which patients presented with cognitive deterioration, gait disturbances, and urinary incontinence together with ventriculomegaly and normal CSF pressure [4]. They emphasized that these patients had treatable dementia and that it was essential to differentiate them from patients with “presenile or senile” dementia or Alzheimer’s disease in whom dilatation of the ventricles could be a consequence of an *exvacuo* brain atrophy [3,4]. After the initial reluctance from neurosurgeons and neurologists to accept this syndrome, it was progressively acknowledged as one treatable cause of dementia. Nevertheless, ongoing controversies about the best screening tools, the most appropriate diagnostic work-up, and the best predictors of outcome in NPH still exist [5]. 

Two patient phenotypes in the NPH syndrome can be found: (1) patients with a known etiology (subarachnoid hemorrhage, head injury, brain tumors, etc.) and (2) those without any recognized cause, labeled as idiopathic NPH (iNPH) [5]. Over the past two decades, Alzheimer’s disease (AD) and Alzheimer’s disease-related dementias have been the target for clinicians and policymakers due to the significant increase in the number of cases as a result of the worldwide growth in the elderly population [6]. Hakim et al. suggested that about 10% of dementia patients could have an iNPH [5]. In a systematic review of the epidemiology of iNPH, Zaccaria et al. reported an incidence ranging from 1.8 to 7.3/100,000 per year and a prevalence ranging from 10 to 29/100,000 [7]. Because iNPH affects <1 in 2000 citizens, according to the European normative, iNPH could be considered a “rare disease”. However, the methodological and clinical heterogeneity of the studies addressing this topic does not allow us to draw adequate conclusions on the true epidemiology of iNPH [7]. Until large population-based studies with homogeneous diagnostic criteria are conducted, the true incidence/prevalence of NPH will not be known, but both are probably largely underestimated. 

The clinical symptoms of iNPH may be improved temporarily by evacuating CSF using a lumbar puncture/drainage; however, the only definite treatment is a CSF diversion with a ventriculoatrial, ventriculoperitoneal, or a lumboperitoneal shunt [8]. The persistent reluctance of many neurologists and neurosurgeons in shunting patients with iNPH is usually justified by the relatively high number of non-responders, the morbidity associated with the surgery, and the lack of “evidence-based” support for clinical decision making. The first influential study on the treatment of NPH was published by Vanneste et al. in 1992 [9]. In this four-center retrospective study and literature review, the authors reported that marked improvements occurred in only 15% of the shunted iNPH patients, and serious adverse events were found in 8.6% of them [9]. They concluded that “The common opinion that improvement of clinical importance in presumed iNPH after a shunt will occur in 35 to 50% is optimistic and may be influenced by a publication bias” [9]. In an editorial commenting on this study, Vanneste remarked that “Most neurosurgeons will not submit these patients to the risks of a shunting procedure because the chance of a postoperative disaster is higher than that of an unexpected surgical success” [10]. Despite very strong opinions such as this one and the lack of randomized-placebo controlled clinical trials that unequivocally justify the benefit of surgery in iNPH patients [11], shunting patients with NPH is a common surgery conducted world-wide with two available prospective multicenter studies showing that most patients who are selected by clinical and neuroradiological findings benefit from surgery with an acceptable risk [12,13,14].

A new wave of therapeutic nihilism has recently resurged. Saper, the Editor-in-Chief of the Annals of Neurology, in an editorial commenting on the American Academy of Neurology guidelines for iNPH released in 2015, asked neurologists to “…seriously consider calling a moratorium on shunting procedures for iNPH until evidence of efficacy is obtained in a rigorous, randomized, placebo-controlled trial” [15]. Despite this highly polarized view, we could use Saper’s own words to define our position as guest editors of this special issue: “but my own reading of the literature and my own clinical experience had indicated that the evidence to support this statement is very weak” [15]. Although not placebo-controlled, the available evidence justifies surgery for iNPH patients. In addition, we believe that not enough clinical equipoise exists among neurologists and neurosurgeons to conduct the “rigorous, randomized, placebo-controlled trial” that Saper claims as a basic requirement to continue shunting patients with iNPH. However, these controversial aspects surrounding the NPH syndrome and its treatment merit careful consideration and will be discussed in depth in this special issue.

Nowadays, three clinical guidelines have been released for the management of NPH patients: (1) the international guidelines published in 2005 [16], (2) the Japanese guidelines written in 2012 [8], and (3) the American Academy of Neurology guidelines released in 2015 [17]. The three guidelines contain recommendations for the diagnosis and management of patients with suspected NPH, but they are quite heterogeneous in their methodological quality when evaluated by using the widely accepted AGREE-II tool [18]. Furthermore, the international guidelines published in 2005 have not been upgraded yet and therefore do not incorporate some recent relevant studies, such as the SINPHONI-2 trial [13]. Many prospective, non-randomized, single-center studies conducted in centers with interest and research programs in NPH have reported above-average good outcomes in iNPH even in patients considered as ‘bad candidates’ for shunting [14,19,20,21]. Despite the usual limitations that uncontrolled clinical trials have, including selection bias and confounding and less generalizable results, the significant differences in these reported outcomes compared to highly-cited studies raises some relevant questions, such as the volume of patients treated by the hospital/individual surgeon and the clinical outcomes for different surgical procedures [22]. In addition, two relevant multicenter studies have reported better-than-expected outcomes. The first was the European multicenter study on iNPH (Eu-iNPH) that enrolled patients who were selected only by clinical and radiological criteria [12]. In this prospective study, 142 patients with iNPH included in 13 European centers were shunted with an adjustable valve [12]. In the Eu-iNPH, 84% of shunted patients showed improvements one year after surgery when evaluated by an ad-hoc NPH scale, and the outcome was also good in 69% of them when evaluated by the less sensitive and motor-oriented modified Ranking Scale [12]. The second study was the SINPHONI-2 trial conducted in Japan [13]. The SINPHONI-2 was a randomized open-label trial, in which two arms were defined to avoid ethical issues: (1) in the first, patients were shunted immediately after randomization with a lumboperitoneal shunt with gravitational control and (2) in the control group, patients underwent the same surgery after a 3-month delay [13]. The primary endpoint was an observed improvement of at least one point on the modified Rankin Scale (mRS) at three months after randomization. Improvements did occur in 65% of the immediately shunted patients compared with only 5% of the patients in the delayed shunted group [13]. 

These new results make it difficult to justify clinical equipoise and therefore the design of randomized placebo-controlled clinical trial as claimed by Saper [15] but should open new research lines dedicated to understanding the reasons why ~20% of patients were non-responders. As remarked by Fasano et al. in a proposed roadmap for research in NPH, “The time has come to start to effectively rethink NPH and determine the many molecular and biological disruptions behind a disorder that, for too long, has remained idiopathic” [23].

Patients with secondary NPH are easily identified in all clinical settings. However, the detection of patients with iNPH remains challenging in an aging society with a high prevalence of cognitive decline and dementia and the associated burden to patients, families, and healthcare services. To improve the treatment of iNPH patients, good screening tests for early detection of potential candidates at the primary care level that might be referred early to neurologists are needed. As remarked by Fasano et al., the need to improve the screening tools and to reach consensus for a universally-accepted and validated NPH scale that covers all the domains of the syndrome exists [23]. A consensus is also needed on the neuroimaging criteria needed to move the screened patients to the category of possible iNPH and therefore refer them to a tertiary center for further evaluations.

At the tertiary centers, we need to clarify whether more sophisticated tests, such as the continuous infusion, Marmarou’s, and tap tests, clinical response to a lumbar drain, intracranial pressure monitoring, and/or more complex and technically-demanding studies, such as compliance and CSF pulse-pressure analysis are needed. These tests might provide additional relevant information for confirming iNPH or predicting the response to a shunt. In this context, we need reproducible biomarkers to detect/exclude concomitant neurodegenerative diseases that can influence a patient’s response to a shunt (Alzheimer’s disease, progressive supranuclear palsy, Lewy body dementia) [23].

Once the patients have been diagnosed with iNPH, we need consensus to define the optimal shunt assembly for treating these patients. We believe that the “one-size-fits-all” approach in some multicenter clinical trials in which a fixed-opening differential-pressure valve (DPV) was implanted [9] or the wide variability in the shunt hardware used in most single-center studies may justify the reported differences in outcomes and the variability in the described complications. The reluctance of the neurosurgical community to incorporate new technologies, such as adjustable-valves and especially, devices for gravitational control, into shunt designs exists because there are no randomized clinical studies that justify these changes. As a result, the neurosurgery community sticks to old DPV designs based on the misleading concept of low-, medium-, and high-pressure DPV “because they still work”. This uncritical thinking needs to be addressed. 

In this focused review for the Journal of Clinical Medicine, we gathered a group of experts with a significant track record in clinical management and research in the field of idiopathic normal pressure hydrocephalus (iNPH) to discuss some of the gaps and ongoing controversies. We believe that evidence-based medicine (EBM) was a paradigm shift in medicine, but we are also concerned with a dogmatic and unrealistic application of EBM to complex diseases, such as iNPH. We fully agree with the statement written in the Trisha Greenhalgh paper on EBM that states, “When medicine is reduced to the dispassionate application of scientific evidence, we will necessarily make worse judgments, not better ones” [24].

We, the guest editors, have enjoyed organizing this Special Issue and are rewarded by the final result of the joint effort. We hope this special issue will accomplish the goal of presenting focused peer-reviewed papers addressing some controversial areas that can be helpful for neurologists, neurosurgeons, and family practitioners when managing iNPH patients.

## References

[1] Penfield W. (1935). The Principles of Physiology Involved in the Management of Increased Intracranial Pressure. Ann. Surg..

[2] Hakim S. (1964). Algunas observaciones sobre la presión del LCR. Sindrome Hidrocefálico en el Adulto con “Presión Normal” del LCR.

[3] Hakim S., Adams R.D. (1965). The special clinical problem of symptomatic hydrocephalus with normal cerebrospinal fluid pressure. Observations on cerebrospinal fluid hydrodynamics. J. Neurol. Sci..

[4] Adams R.D., Fisher C.M., Hakim S., Ojemann R.G., Sweet W.H. (1965). Symptomatic Occult Hydrocephalus with “Normal” Cerebrospinal-Fluid Pressure. A Treatable Syndrome. N. Engl. J. Med..

[5] Hakim C.A., Hakim R., Hakim S. (2001). Normal-pressure hydrocephalus. Neurosurg. Clin. N. Am..

[6] National Academies of Sciences, Engineering, and Medicine (2018). Future Directions for the Demography of Aging: Proceedings of a Workshop.

[7] Zaccaria V., Bacigalupo I., Gervasi G., Canevelli M., Corbo M., Vanacore N., Lacorte E. (2020). A systematic review on the epidemiology of normal pressure hydrocephalus. Acta Neurol. Scand..

[8] Mori E., Ishikawa M., Kato T., Kazui H., Miyake H., Miyajima M., Nakajima M., Hashimoto M., Kuriyama N., Tokuda T. (2012). Guidelines for management of idiopathic normal pressure hydrocephalus: Second edition. Neurol. Med. Chir..

[9] Vanneste J., Augustijn P., Dirven C., Tan W.F., Goedhart Z.D. (1992). Shunting normal-pressure hydrocephalus: Do the benefits outweigh the risks? A multicenter study and literature review. Neurology.

[10] Vanneste J.A.L. (1994). Three decades of normal pressure hydrocephalus: Are we wiser now?. J. Neurol. Neurosurg. Psychiatry.

[11] Esmonde T., Cooke S. (2002). Shunting for normal pressure hydrocephalus (NPH). Cochrane Database Syst. Rev..

[12] Klinge P., Hellstrom P., Tans J., Wikkelso C., European iNPH Multicentre Study Group (2012). One-year outcome in the European multicentre study on iNPH. Acta Neurol. Scand..

[13] Kazui H., Miyajima M., Mori E., Ishikawa M., SINPHONI-2 Investigators (2015). Lumboperitoneal shunt surgery for idiopathic normal pressure hydrocephalus (SINPHONI-2): An open-label randomised trial. Lancet Neurol..

[14] Poca M.A., Solana E., Martinez-Ricarte F.R., Romero M., Gandara D., Sahuquillo J. (2012). Idiopathic normal pressure hydrocephalus: Results of a prospective cohort of 236 shunted patients. Acta Neurochir. Suppl..

[15] Saper C.B. (2016). The Emperor has no clothes. Ann. Neurol..

[16] Marmarou A., Black P., Bergsneider M., Klinge P., Relkin N. (2005). Guidelines for management of idiopathic normal pressure hydrocephalus: Progress to date. Acta Neurochir. Suppl..

[17] Halperin J.J., Kurlan R., Schwalb J.M., Cusimano M.D., Gronseth G., Gloss D. (2015). Practice guideline: Idiopathic normal pressure hydrocephalus: Response to shunting and predictors of response: Report of the Guideline Development, Dissemination, and Implementation Subcommittee of the American Academy of Neurology. Neurology.

[18] Brouwers M.C., Kho M.E., Browman G.P., Burgers J.S., Cluzeau F., Feder G., Fervers B., Graham I.D., Grimshaw J., Hanna S.E. (2010). AGREE II: Advancing guideline development, reporting and evaluation in health care. Can. Med Assoc. J..

[19] Eide P.K., Sorteberg W. (2010). Diagnostic intracranial pressure monitoring and surgical management in idiopathic normal pressure hydrocephalus: A 6-Year review of 214 patients. Neurosurgery.

[20] Poca M.A., Mataro M., del Mar M.M., Arikan F., Junque C., Sahuquillo J. (2004). Is the placement of shunts in patients with idiopathic normal-pressure hydrocephalus worth the risk? Results of a study based on continuous monitoring of intracranial pressure. J. Neurosurg..

[21] Poca M.A., Mataro M., Matarin M., Arikan F., Junque C., Sahuquillo J. (2005). Good outcome in patients with normal-pressure hydrocephalus and factors indicating poor prognosis. J. Neurosurg..

[22] Gruen R.L., Pitt V., Green S., Parkhill A., Campbell D., Jolley D. (2009). The effect of provider case volume on cancer mortality: Systematic review and meta-analysis. CA Cancer J. Clin..

[23] Fasano A., Espay A.J., Tang-Wai D.F., Wikkelsö C., Krauss J.K. (2020). Gaps, Controversies, and Proposed Roadmap for Research in Normal Pressure Hydrocephalus. Mov. Disord..

[24] Greenhalgh T. (2012). Why do we always end up here? Evidence-based medicine’s conceptual cul-de-sacs and some off-road alternative routes. J. Prim. Health Care.

